# A Broadband Mid-Infrared Trace Gas Sensor Using Supercontinuum Light Source: Applications for Real-Time Quality Control for Fruit Storage

**DOI:** 10.3390/s19102334

**Published:** 2019-05-21

**Authors:** Khalil Eslami Jahromi, Qing Pan, Amir Khodabakhsh, Cor Sikkens, Paul Assman, Simona M. Cristescu, Peter M. Moselund, Maxime Janssens, Bert E. Verlinden, Frans J. M. Harren

**Affiliations:** 1 Trace Gas Research Group, Institute of Molecules and Materials, Radboud University, 6525 AJ Nijmegen, The Netherlands; kh.eslami@science.ru.nl (K.E.J.); A.Khodabakhsh@science.ru.nl (A.K.); C.Sikkens@science.ru.nl (C.S.); p.assman@science.ru.nl (P.A.); s.cristescu@science.ru.nl (S.M.C.); F.Harren@science.ru.nl (F.J.M.H.); 2 NKT Photonics A/S, Blokken 84, DK-3460 Birkeroed, Denmark; pmm@nktphotonics.com; 3 Flanders Center of Postharvest Technology, Willem de Croylaan 42, 3001 Heverlee, Belgium; maxime.janssens@vcbt.be (M.J.); bert.verlinden@vcbt.be (B.E.V.)

**Keywords:** mid-infrared, broadband, supercontinuum, trace gas sensing, multi-species, real-time monitoring, fruit storage

## Abstract

We present a fully integrated and transportable multi-species trace gas sensor based on a mid-infrared (MIR) supercontinuum light source. The high brightness (surpassing synchrotron) and ultra-broad spectral bandwidth (2–4 μm) of this light source allows simultaneous detection of multiple broadband absorbing gas species. High sensitivity in the sub-ppmv level has been achieved by utilizing an astigmatic multipass cell. A grating-based spectrometer at a scanning rate of 20 Hz is developed employing a balanced detection scheme. A multi-component global fitting algorithm is implemented into a central LabVIEW program to perform real-time data analysis. The obtained concentration values are validated by the standard gas chromatography mass spectrometry (GC-MS) method. Field application of the sensor for quality control of stored fruits at a small scale is demonstrated, involving the detection of ethylene, ethanol, ethyl acetate, acetaldehyde, methanol, acetone, and water simultaneously. The sensor also shows promising potentials for other applications, such as environmental monitoring and biomedical research.

## 1. Introduction

The high economic value of sustainable agriculture has a direct impact on society. While the demand for agro-products is increasing because of global population growth, it is also becoming more and more important to avoid food spoilage/degradation. The Food and Agricultural Organization (FAO) of the United Nations estimates that, globally, 33% of the edible parts of food produced for human consumption are lost or wasted. Worldwide, 1.6 billion tons of fruit and vegetables are produced, from which 120 million tons are lost during the post-harvest process, i.e., losses due to spoilage/degradation during handling, storage and transportation between farming and distribution [1]. One efficient way to reduce losses in commercial-scale storage is to store agro-products in a dynamically controlled atmosphere (DCA) [2] and continuously monitor the emitted volatile species [3]. Unfortunately, challenges arise, as there is currently no equipment that can simultaneously and reliably measure multiple volatiles with sufficient sensitivity in a cost-effective way.

Nowadays, there are various types of detection methods that are at the frontier of trace gas sensing research [4,5,6,7,8]. In particular, MIR laser absorption spectroscopy provides a unique opportunity for fast, sensitive, and non-destructive detection [9,10]. On one hand, the rotational-vibrational transitions of molecular species generally show characteristic absorption patterns in the so-called “MIR fingerprint region” (approximately 2–15 μm). These fundamental transitions are generally significantly stronger than the overtone bands in the near-infrared region by orders of magnitude. On the other hand, the fast advancement of bright, stable, and compact laser sources in the MIR region facilitates the detection of these patterns at high speed and low cost, depending on the requirements for various field applications.

However, the limited mode-hop-free tuning over a wide wavelength range of traditional lasers only allows the measurement of individual absorption lines of some molecular species, inevitably limiting the detection of larger molecules, which typically have overlapping rotational absorption lines within a vibrational band. Such overlapping lines generally result in broad absorption features, thus requiring a broadband light source to detect. Furthermore, it is even more challenging to detect multiple overlapping molecular species simultaneously. Traditional MIR broadband sources such as Globar may be considered, but the low spectral power density and poor directionality of these sources limit their practical applications.

Recently, frequency combs and supercontinuum (SC) sources are emerging as a new generation of broadband light sources for laser absorption spectroscopy [11,12,13]. While novel comb-based detection techniques such as dual-comb spectroscopy [14,15] and Vernier spectroscopy [16] have successfully demonstrated high spectral resolution and detection sensitivity, the instrumental complexity and cost are still far beyond the techno-economic constrains for field applications. The SC sources, on the other hand, are less expensive alternatives, although they do not provide high shot-to-shot coherency of the frequency combs. Since high coherency is not a prerequisite for direct absorption spectroscopy, the phase noise does not require sophisticated opto-electronic control while generating the SC beam. The relief of this technical constrain has a direct impact to the overall system complexity, making the SC sources very promising for broadband MIR spectroscopy.

In the present contribution, we demonstrate the development, validation, and application of a transportable MIR SC-based trace gas sensor for parallel detection of multiple gas species. We explore the instrumental sensitivity from the parts-per-million gas volume mixing (ppmv) region down to the parts-per-billion (ppbv) limit for broadband absorbing gases. A global fitting algorithm is developed and implemented to a central LabVIEW program for real-time data analysis. Practical issues such as water interference are addressed, and the obtained concentration values are compared with GC-MS results, showing high promise for future multi-species trace gas analysis in other application fields, even beyond fruit storage.

## 2. Materials and Methods

### 2.1. The Targeted Volatile Species

Gas sensing in storage rooms of fresh fruits is, in general, a common practice. Since the 1950s, controlled concentrations of oxygen and carbon dioxide have been applied to extend the storage time of various agro-products. Such interactive storage concepts are mainly adopted to enable storage at the lowest possible oxygen concentration without inducing fermentation. In order to obtain information about the physiological and pathological status of the stored agro-products, it is a promising way to monitor the product-produced volatiles in the storage atmosphere, leading to a better control over the quality and thus mitigating losses.

In the field of controlled atmosphere, the respiration quotient (RQ), which is defined as the ratio between the produced carbon dioxide and consumed oxygen, is traditionally adopted as a parameter for adjusting the oxygen concentration in the storage rooms. Recent research also focuses on various volatiles released from the stored products, including ethylene, a marker for ripening [17], and ethane, an indicator of damages such as chilling injury [18]. In addition, ethanol, acetaldehyde, and ethylacetate are well known volatile markers for fermentation [19], and methanol and acetone may serve as rotting process indicators [20]. More specifically, the development of fungal infections (potentially dangerous for the consumer’s health) during storage may also be detected by rotting markers in combination with low levels of fermentation markers. It is therefore important to monitor such volatile markers, not only for food quality, but also for food safety control.

The aforementioned volatiles are often released in tiny amounts, hence requiring long accumulation periods to obtain detectable signals. Note that during such accumulation periods, the concentrations of many compounds inside and outside the agro-products can change, thereby possibly influencing the metabolism processes. It is therefore also important to continuously monitor multiple gas species in real-time in order to obtain comprehensive information to prevent further unwanted processes at the early stage.

### 2.2. The Supercontinuum Light Source

**Figure 1:**
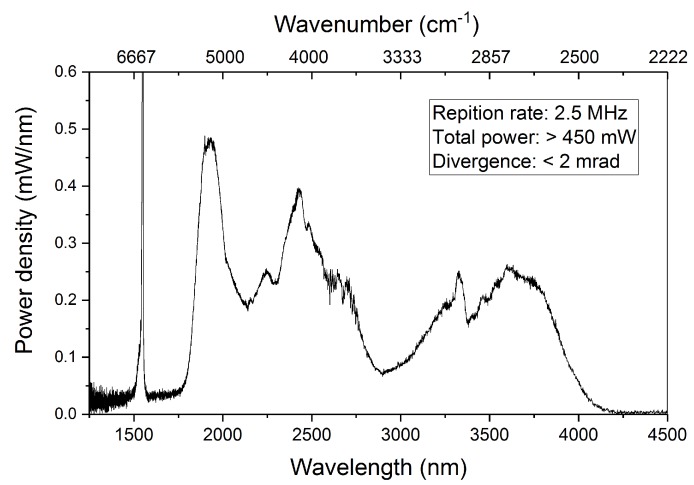
Spectral power density of the SC light source integrated into the gas sensor.

The breakthrough of SC generation in the MIR region is more recently demonstrated in comparison to the SC in the near-infrared region [12,21]. In general, SC light can be generated by broadening the spectrum of a pulsed pump laser in a nonlinear optical fiber through complex processes such as soliton fission and Raman scattering. For the present work, a MIR SC source (SuperK MIR, NKT Photonics Southampton, UK) is integrated into the sensor system. Unlike traditional infrared lamps, this light source shows high directionality with a beam diameter of ~3 mm and a divergence of less than 2 mrad. It covers a broad spectral window from 1500 nm up to 4200 nm, as shown in Figure 1. The spectrum was measured by using a calibrated spectrum analyzer (Mozza, Fastlite, Antibes, France). The total optical power is above 450 mW at a repetition rate of 2.5 MHz. In particular, the averaged spectral power density is ~0.18 mW/nm in the 3000–4000 nm window, where characteristic molecular vibrations such as C-H stretching are present. Note that the brightness of this source even surpasses that of a typical synchrotron beamline [22]. The combination of high spatial coherence, wide spectral coverage, and high spectral power density in a compact configuration makes this light source suitable for a transportable and broadband trace gas sensor, which can detect multiple gas species in parallel.

### 2.3. The Optical Setup

**Figure 2:**
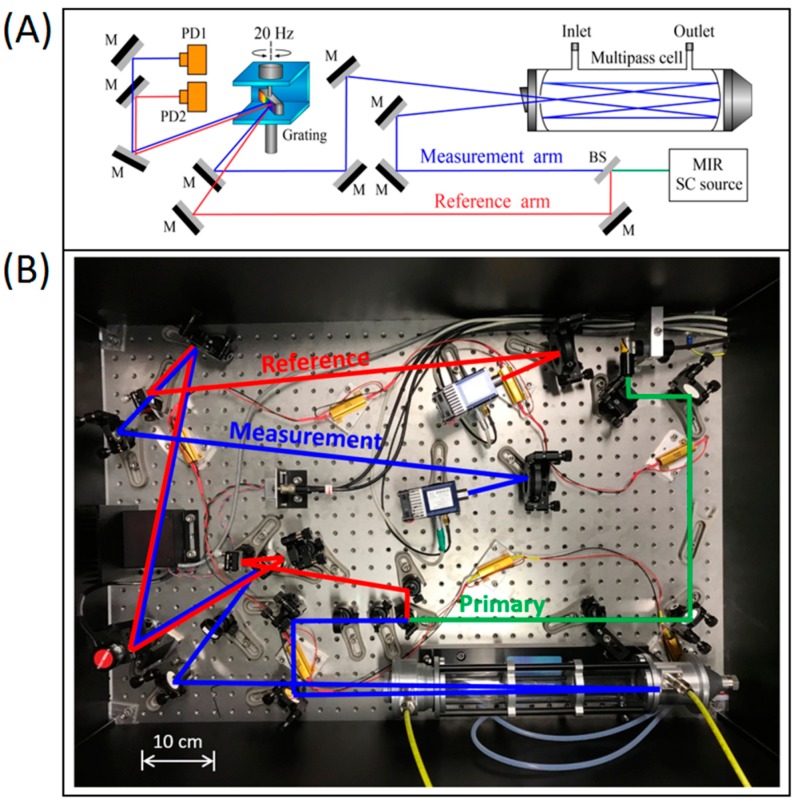
(**A**) Simplified schematic representation of the optical setup (M: mirror, BS: beam splitter, PD: photodetector). (**B**) Top view of the optical system. Note that the primary SC beam (highlighted in green) is split into the measurement (blue) and reference (red) beams.

The broadband nature of the SC light source has opened up new possibilities for multi-species trace gas sensing based on direct absorption spectroscopy. In order to improve the sensitivity, we combined the SC source with an astigmatic multipass cell (AMAC-76, Aerodyne Research, Billerica, MA, USA) which can provide an effective optical path-length of 76 m. A simplified schematic representation of the setup is shown in Figure 2A. A balanced detection technique is implemented by splitting the primary SC beam into a measurement arm and a reference arm. The beam in the measurement arm is coupled to the multipass cell to measure the absorption features of the targeted gas species. The beam in the reference arm bypasses the multipass cell to counterbalance potential spectral and power drifts of the SC source. Both the measurement and reference beams are aligned towards a fast-scanning diffraction grating (450 grooves/mm, GR1325-45031, Thorlabs GmbH, Dachau, Germany) with the same horizontal incident angle but at different heights on the grating. The grating is mounted on a galvo scanner (GVS011/M, Thorlabs GmbH, Dachau, Germany) driven by a sinusoidal wave at 20 Hz in order to sweep the dispersed SC light. Two thermoelectrically cooled HgCdTe photodetectors, featuring 10^11^ cm·Hz^1/2^/W specific detectivity (PVI-4TE-4, VIGO System SA, Ożarów Mazowiecki, Poland), are utilized to detect the spectrally resolved main and reference signals. This detection scheme allows a continuous normalization of the sample spectrum with respect to the background spectrum, enhancing the sensitivity and long-term stability.

Figure 2B shows a top view of the optical setup deployed on a 90 × 60 cm^2^ optical breadboard. In practice, a wedged and uncoated CaF_2_ window is inserted into the primary beam path to reflect a minority portion as the reference, while the transmitted majority portion (i.e., the measurement beam) is guided into the multipass cell. Note that the multipass cell has ~96% power attenuation due to the reflection loss at the front window and the limited reflectivity of the cell mirrors over the broad spectral range of the SC source. The overall effect is that the reference and measurement beams have comparable power on the grating. Since the two incidence angles of the measurement and reference beams are identical with respect to the grating surface in the horizontal plane, the diffracted signals also travel along the same horizontal direction. Two cylindrical mirrors are utilized to focus the diffracted signals onto the two photodetectors, which are synchronized. The spectral measurement time is 50 ms, limited by the grating scanning speed. A spectral window of ~460 cm^−1^ (2700–3160 cm^−1^) is covered, limited by the maximum scanning angle of the grating. A spectral resolution of ~3 cm^−1^ is obtained, verified by measuring the characteristic methane absorption patterns, as shown in Figure S1 in the Supplementary Information (SI).

### 2.4. The Integrated System

**Figure 3:**
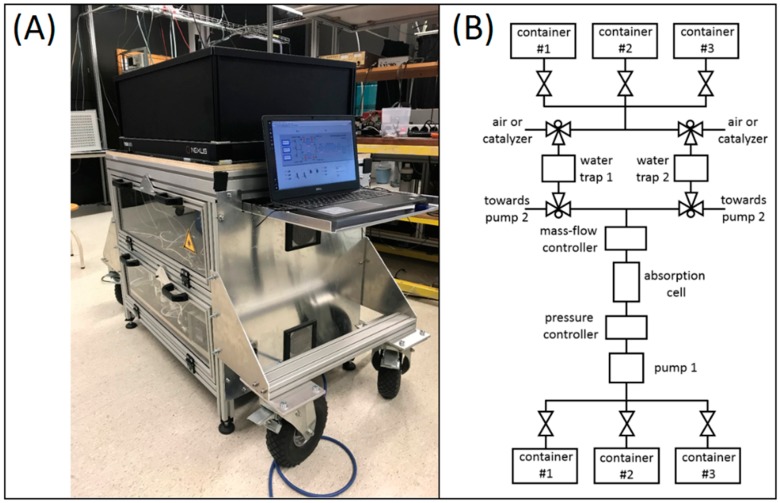
(**A**) Overview of the transportable and integrated gas sensing platform. (**B**) Schematic overview of the gas handling system.

Figure 3A shows an overview of the fully integrated system composed of three main compartments. The optical compartment situating on the top is completely shielded and is kept in 21 °C in order to improve the thermal stability. The optoelectronic components are located in the middle compartment, including the data acquisition system (USB-6211, National Instruments Netherlands BV, Woerden, The Netherlands) and the drivers for the SC source, the galvo scanner, and the photodetectors. All the hardware components associated with gas flow are located in the bottom compartment. The sensor platform consumes ~550 W when running at full capacity, and is controlled by a central laptop for automated operation.

For applications involving small-volume (≤ 300 L) fruit storage containers, a closed-loop gas flow configuration is applied, allowing gas recycling. More specifically, the gas extracted from the storage container can flow back after the non-destructive optical analysis in the absorption cell (0.5 L volume). In this way, disturbance to the storage atmosphere is minimized, mimicking long-term storage conditions. A schematic representation of the gas handling system is shown in Figure 3B, and the associated LabVIEW-based monitoring program is shown in Figure S2 (SI).

To reduce the adsorption effect of polar molecules on wall materials, polytetrafluoroethylene tubes (6 mm outer diameter, Polyfluor Plastics B.V., Breda, The Netherlands), valves (PKV series, Takasago Fluidic Systems, Nagoya, Japan), and membrane pumps (N920G and N86KN.18, KNF-Verder B.V., Vleuten, The Netherlands) are utilized for the gas handling system. A pressure controller (EL-PRESS, Bronkhorst Netherland B.V., Veenendaal, The Netherlands) and a mass-flow controller (EL-FLOW Prestige, Bronkhorst Netherland B.V., Veenendaal, The Netherlands) are also integrated to ensure a stable measurement condition (i.e., 900 mbar and 5 L/h flow rate). Figure S3 (SI) shows the integrated gas handling system.

As water vapor is known to be a common interfering species for absorption spectroscopy in the present spectral window, a water trapping system is developed to physically reduce the humidity of the gas flow through direct contact with a thermoelectrically cooled freezing surface. Since the vapor pressure of the target volatiles (e.g., ethylene) is substantially higher than the water counterpart, as shown in Figure S5 (SI), this cooling process would not significantly disturb the phase of the target volatiles. The possibility of adsorption due to temperature change cannot be completely excluded. However, this effect is not significant in the low ppmv region, confirmed by evaluating a calibrated ethanol source of 5 ppmv (*vide infra*).

In practice, this water trap consists of two cooling chambers controlled by independent Peltier elements, whose polarity can be swapped automatically. This configuration ensures the water trap can operate continuously when one chamber is cooling and another one is in the de-icing process. In order to further reduce the temperature, a two-stage cooling method is applied by circulating anti-freezing liquid between the two cooling layers, effectively achieving −16 °C when operating from room temperature. Furthermore, two peristaltic water pumps are integrated to physically remove liquid water (melted ice) more efficiently from the cooling chamber. The developed water trapping system is shown in Figure S4 (SI).

### 2.5. Real-Time Data Analysis

In order to continuously monitor the concentration of the targeted volatiles, a real-time data analysis algorithm was developed and implemented to the central LabVIEW program. This algorithm takes advantage of the broadband spectral range to perform a global fitting, instead of focusing on a specific narrow spectral feature, enhancing the overall precision for multi-species detection.

In general, the measured absorbance spectrum can be represented by a one-dimensional matrix containing *n* non-negative spectral elements. This matrix, i.e., ***M*_*n*×1_**, can be linearly decomposed into a summation of multiple single-species absorbance patterns (of a unit concentration, e.g., per ppmv) weighted by their individual concentration ***C*_*k*×1_** (*k* represents the number of target gas species). The reference absorbance of each individual species can be calculated using different databases (e.g., HITRAN [23] and PNNL [24]), or can be experimentally measured in advance using calibrated gases of known concentrations. To simplify the calibration process, we chose the latter option and established a reference database, i.e., ***R*_*n*×*k*_**, accordingly. Hence, it is an inverse problem to calculate the concentration matrix (***C*_*k*×1_**) based on the measured absorbance (***M_n_*_×1_**) and the known reference (***R_n_*_×*k*_**) by globally minimizing the root-mean-square (RMS) error (***E*_*n*×1_**) in the following mathematical expression:

***M*_*n*×1_** = ***R*_*n*×*k*_*C*_*k*×1_** + ***E*_*n*×1_** (1)

In the above expression, a broadband absorbance spectrum can be covered by taking a large number of spectral elements *n*. This method is particularly beneficial to decompose (partially) overlapping features. In practice, a priori knowledge of the number of target gas species and their absorbance patterns is mandatory for the algorithm. However, the reference database ***R*_*n*×*k*_** can be always extended by adding more components.

**Figure 4:**
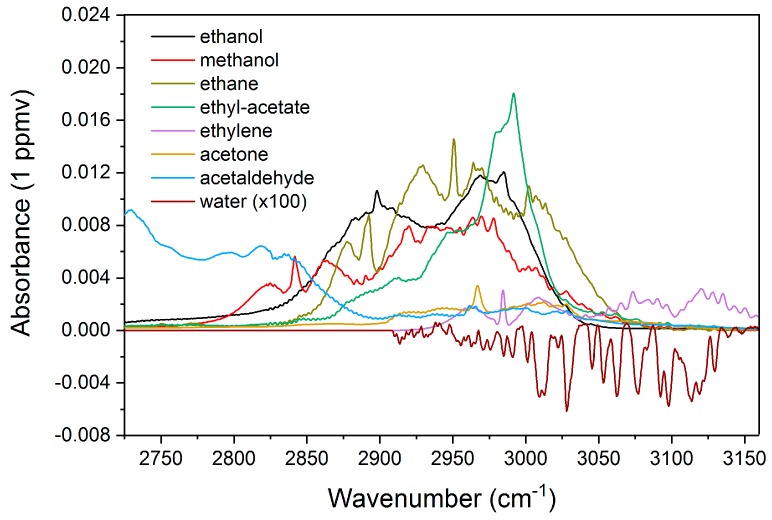
The reference absorbance (base-10) database at 1 ppmv level, including the targeted gas species. Note that water absorbance is inverted and re-scaled for clarity.

In the present work, seven volatiles highlighted in Section 2.1 are considered in the reference database, including ethanol, methanol, ethane, ethyl-acetate, ethylene, acetone, and acetaldehyde. Calibrated gas bottles (Linde Gas Benelux B.V., Schiedam, The Netherlands) of known concentration have been utilized for benchmarking. The individual absorbance spectra corresponding to 1 ppmv are re-scaled from the signals measured at 100 ± 2 ppmv level. This method maximizes the signal-to-noise ratio of the developed reference database, and can therefore minimize the systematic errors at the subsequent global fitting stage. Furthermore, the water absorbance pattern is also included in the database to account for residual water vapor in the gas sample after the water trap. An overview of the absorbance (base-10) database is presented in Figure 4. Note that the water absorbance spectrum is inverted and is magnified by a factor of 100 (i.e., ~100 ppmv) for clarity.

For practical applications, the spectral calibration of the grating-based spectrometer may drift due to mechanical disturbance, deteriorating the validity of the reference database. In order to counterbalance this drift, an additional fitting step is applied by shifting the reference spectra by ±2 cm^−1^ in small steps. The corresponding RSM errors are evaluated, and the fitted result with the minimum error is adopted. This method is implemented in the central LabVIEW program to correct, in real-time, potential incorrect calibration of the spectrometer.

Since water, a common interfering species, can reach ~1000 ppmv concentration level even after the water trap, it is particularly important to minimize the water-induced fitting error prior to calculating the concentrations of the targeted species, which are typically at low ppmv or sub-ppmv levels.

In practice, the measured spectral window is divided into two sub-windows, and a two-stage fitting method is applied. This method firstly performs a global fit in the 2900–3160 cm^−1^ window by taking the concentrations of water and other volatiles of interest, especially ethylene, as free parameters. This fitting window is specifically chosen because the absorption of water and ethylene is negligible below 2900 cm^−1^. The obtained water and ethylene concentrations are then treated as fixed parameters for a second round of fitting in the 2725–3000 cm^−1^ window, where the concentrations of other target volatiles are determined based on the minimum RMS error criteria.

**Figure 5:**
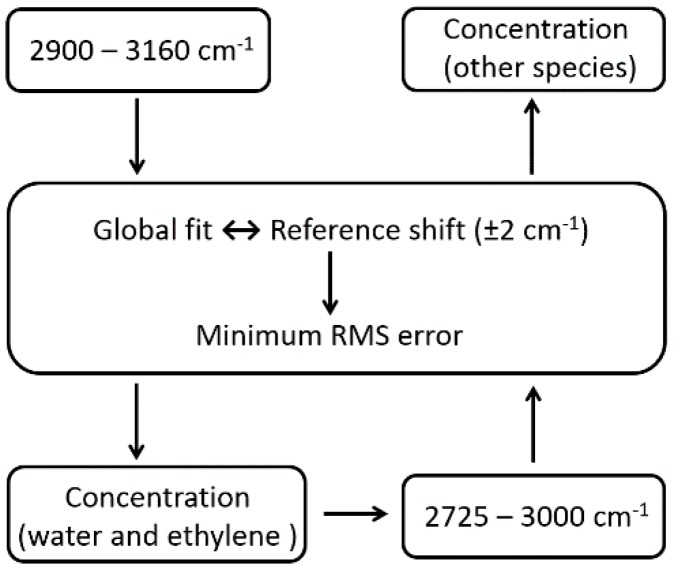
Procedure overview of a two-stage global fitting method for concentration calculation.

Figure 5 summarizes the procedures of the two-stage fitting method. Note that ethylene concentration is also chosen as a fixed parameter for the second fitting round, as the absorption features of ethylene and water are mostly overlapping in the 2900–3160 cm^−1^ window.

### 2.6. Pear Storage and TD-GC-MS Analysis of Storage Atmosphere Volatiles

Conference pears were harvested on 24^th^ of August 2018 and stored at −1 °C. After three weeks of storage in regular air, the pears were transferred into an air-tight polypropylene fruit storage container (≤300 L) in which the atmosphere was controlled using a DCA protocol according to [25]. Carbon dioxide concentration was maintained at 0.7 kPa while oxygen concentration was allowed to vary between 3 and 0.2 kPa, according to measured RQ values in the DCA protocol.

After 5.5 months of storage, the storage atmosphere inside the container was sampled in Tedlar bags (5 L, MediSense, Winschoten, The Netherlands). Multisorbent thermal desorption (TD) tubes of Carbopack B/Carbopack X/Carbosieve-III (GERSTEL GmbH & Co. KG, Mülheim an der ruhr, Germany) were used to collect the volatiles for desorption. An air sampling pump (Gilian GilAir Plus, Sensidyne, LP, St. Petersburg FL, USA) was programmed to draw in 1000 mL of the gaseous sample from the Tedlar bag into the TD tube. The pump was calibrated using a flow meter (Defender 530+, MesaLabs, Butler, PA, USA). Before the air passed the TD tube, it passed a glass tube filled with glass beads held in ice to reduce the water concentration.

Volatile analysis was done using a GC (Agilent 7890A, Agilent Technologies, Santa Clara, CA, USA) combined with a mass spectrometer (Agilent 5973C VL MSD Triple Axis Detector, Agilent Technologies, Santa Clara, CA, USA). The GC-MS was equipped with a thermal desorption unit, (CIS 4, GERSTEL GmbH & Co.KG, Mülheim an der ruhr, Germany) which was cooled with liquid nitrogen. The desorption tubes were placed inside the TD unit and were heated to release all volatiles adsorbed. Subsequently, these volatiles were trapped again in a confined space using liquid nitrogen before being injected into GC. Before the desorption step, the tubes were purged for 5 min at 10 °C to remove excess water. A temperature program from 10 °C to 270 °C (hold 4 min) at a rate of 100 °C/min was used on the TD unit to desorb the adsorption tubes. Helium was used as a carrier gas at a flow rate of 50 mL/min, and a temperature program from −50 °C to 250 °C (hold 5 min) at a rate of 12 °C/sec was used for the CIS. A 25 m Pora Bond Q column (CP7348PT) with a diameter of 0.25 mm and film thickness of 3 µm (Agilent J&W GC columns) was used. The oven temperature was set at 35 °C (hold 0 min) to 170 °C at 10 °C/min (hold 0 min) and 20 °C/min to 250 °C (hold 5 min) at a constant carrier gas flow of 1 mL/min. A full scan, 12–350 amu at 4.17 scan/sec was carried out on the MS-detector.

## 3. Results

As highlighted in the introduction section, the broadband trace gas sensor is particularly designed for multi-species detection for quality control of agro-products. Specifically, we focus on real-time pear-storage conditions. Since the storage atmosphere is typically complex (and thus not completely known), we start the discussion of calibrated gas mixtures of known concentrations for benchmarking purpose.

We firstly evaluate the performance of the trace gas sensor by measuring a single target species, i.e., calibrated 5 ± 0.1 ppmv ethanol in nitrogen. A satisfactory result of 4.87 ± 0.32 ppmv has been obtained based on 100 independent measurements (30 s averaging time for each measurement). This result also suggests that adsorption due to the water trap (cooling chamber) is not significant. Typical measured and fitted absorbance spectra are presented in Figure S6 in the Supporting Information. Since the absorption strength of other species of interest (e.g., methanol) is comparable with that of ethanol, the detection sensitivity would be also comparable for single species measurement.

Challenges arise when detecting multiple gas species which have overlapping absorption patterns in an unknown atmosphere condition. We address this issue by measuring a gas mixture containing multiple targeted species and four additional compounds unknown to the reference database, i.e., 1-propanol, 2-butanone, propylene, and propionaldehyde. To explore the detection limit, the concentration of each species was diluted to ~390 ppbv except for ethylene, whose concentration was ~19.5 ppmv. This dramatic concentration contrast was applied because ethylene concentration was known to be at significantly higher levels in pear storage containers. More importantly, this contrast allowed us to test the system’s capability of detecting small concentration values out of a dominating ethylene signal.

Table 1 shows the measured concentration values for the complex gas mixture. The dominating ethylene (~19.5 ppmv) signal is successfully detected, whereas other volatiles in the ppbv level are mostly over-estimated. This result can be rationalized by the contribution of the four additional species absent in the reference database. Remarkably, the uncertainty in the order of 100 ppbv suggests that sub-ppmv sensitivity can be achievable, reflecting the potential to detect multiple gas species in parallel in a complex atmosphere condition.

**Figure 6:**
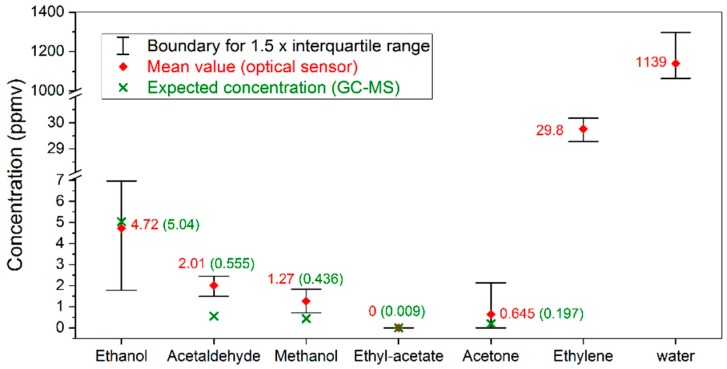
Comparison of the obtained concentration values using the optical method (red) and the TD-GC-MS method (green).

In order to further explore the application potential to real storage conditions, we applied the sensor to a commercial storage container (300 L), in which pears were stored in a DCA environment of 0.7% CO_2_ level. TD-GC-MS method was also applied for comparison. The obtained results are summarized in Figure 6.

Each mean value point for the optical sensing method presented in Figure 6 is derived from 57 measurements, each averaged for one minute. The associated outlier represents the 1.5 interquartile range. The data are compared with TD-GC-MS results (in green), which are obtained by the average of two gas samples based on TD injection. The TD-GC-MS was not able to provide ethylene and water concentrations, due to limitations of the instrument. However, since the absorbance spectra are mostly dominated by ethylene and water, detecting these two species is not as challenging as detecting the other volatiles, which will be focused on in the next discussion section.

## 4. Discussion

In this section, we explore the capability and limitations of the multi-species trace gas sensor by evaluating the data presented in Figure 6 in more detail.

**Figure 7:**
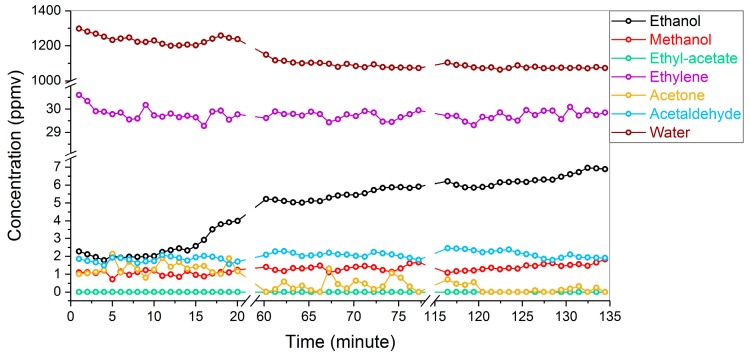
Time-dependent representation of the obtained concentration values of multiple gas species based on the same pear-storage container. The breaking of the time axis is due to an alternating operation configuration to measure two different storage containers. For clarity purposes, the data of the other container are not shown.

Firstly, the obtained mean value of ethanol concentration matches the TD-GC-MS result. However, the associated outlier is seemingly much higher comparing to the uncertainty levels of the other species. In fact, as shown in Figure 7, this wide outlier results from a slow ethanol emission process during our measurements. Note that the samples for the TD-GC-MS were only manually collected by the end of the optical measurement, thus reflecting only the validity of the last few data points in the ethanol evolution curve. Analogously, a concentration reduction down to the detection limit of the optical sensor is also observed for acetone, rationalizing the relatively high upper boundary of the associated outlier in Figure 6. These results demonstrate the advantage of automated and parallel measurement of gases in a continuous flow, comparing to traditional static measurement based on manual sampling.

**Figure 8:**
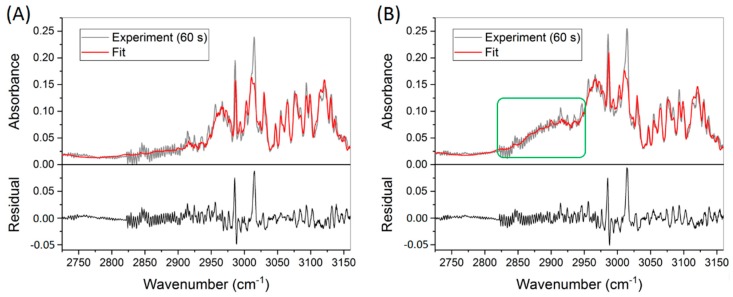
Measured (grey) and fitted (red) absorbance spectra associated with the first (**A**) and last (**B**) concentration data points in Figure 7. The residuals are included on the bottom. The green square in (**B**) highlights an increased ethanol concentration. A Savitzky–Golay filter is applied in the 2725–2821 cm^−1^ window in order to minimize the etalon effect due to optical elements.

Figure 8A,B show the absorbance spectra associated with the first and last measurements of Figure 7, respectively. The sharp peak at ca. 2987 cm^−1^ is assigned to ethylene, and the other peaks at higher wavenumbers are dominated by both ethylene and water. Remarkably, as highlighted by the green square in Figure 8B, a broad absorbance band is clearly observed in the 2850–2950 cm^−1^ region as a result of ethanol emission. This result demonstrates the advantage of global fitting based on a wide spectral window rather than a particular wavenumber to measure various volatiles in parallel.

For the detection of acetaldehyde and methanol, the obtained stable concentration shows good precision and reproducibility with a standard deviation of ~220 ppbv, suggesting sub-ppmv sensitivity. The over-estimation of these two species is possibly due to the incompleteness of the reference database, as the residual plots in Figure 8 show an intense peak around 3016 cm^−1^ attributed to an unknown species. In addition, sample losses due to the TD tubes and the injection process prior to the GC measurements may also contribute to the discrepancy between these two detection methods.

As the expected concentration of ethyl acetate (i.e., 9 ppbv, measured by the TD-GC-MS) is significantly below the detection limit of the optical sensor, the global fitting algorithm consequently treats this species as a non-contributing component for the overall absorbance spectrum, leading to a “zero” output in order to achieve the minimum RMS error. The reproducibility of this output also demonstrates good selectivity, which is important for large fruit storage facilities to avoid false alarms.

In order to check the long-term reproducibility, two independent measurements on the same storage container were performed in two consecutive weeks. In order to test the influence of the overwhelming ethylene signal on detecting the other volatiles, three additional containers with different ethylene concentration were measured. The results are summarized in Figure S7 in the Supporting Information. A statistical evaluation shows that the sensor cannot retrieve meaningful concentration values when ethylene is above 50 ppmv whereas the other volatiles are in the <500 ppbv regime. When ethylene concentration is relatively lower, reproducible results have been obtained. A side-by-side comparison with the TD-GC-MS data shows that the optical sensor mostly over-estimate acetaldehyde and methanol. However, the relative concentration, in comparison to other volatiles, is still in general agreement.

For future development, an expansion of the reference database would be beneficial to enhancing the overall accuracy, requiring a systematic investigation of the fruit storage atmosphere in more detail by applying more complementary detection methods. This target-oriented assessment is beyond the scope of the present discussion, which mainly focuses on the introduction of a new SC-based multi-species gas sensing technique. Applications involving a variety of agro-products including pears, apples, blueberries, and potatoes will be covered in future publications.

## 5. Conclusions

In summary, we have presented the first study to investigate multi-species trace gas sensing using a broadband MIR SC source for fruit storage applications. A spectrally resolved balanced detection technique is applied involving a scanning grating spectrometer at 20 Hz. A global fitting algorithm is implemented for real-time data analysis. The target is to continuously and automatically monitor multiple volatiles emitted from agro-products during storage. We have evaluated the capability and limitations of the system by testing, in realistic storage conditions, in terms of sensitivity, selectivity, and reproducibility. The detection limit is explored for applications with dramatic concentration contrast of at least two orders of magnitude for various gases. The obtained results show a promising future for practical fruit storage and other applications requiring multi-species and real-time trace gas sensing.

## Figures and Tables

**Table 1:** Concentration overview of the gas mixture diluted from a calibration gas source containing nine species (second column) in comparison with the measurement results (fourth column).

Compound Name	Calibrated Concentration (ppmv)	Diluted Concentration (Expected, ppmv) *	Measured Concentration (ppmv) **
Ethylene	5000 ± 25	~19.5	19.5 ± 1.73
Ethanol	100 ± 0.5	~0.39	1.55 ± 0.38
Acetaldehyde	100 ± 5	~0.39	1.82 ± 0.64
Methanol	100 ± 1	~0.39	0.71 ± 0.19
Ethyl-acetate	100 ± 1	~0.39	0.60 ± 0.10
Acetone	100 ± 0.5	~0.39	0
1-propanol	100 ± 1	~0.39	N.A.
2-butanone	100 ± 5	~0.39	N.A.
Propylene	100 ± 1	~0.39	N.A.
Propionaldehyde	100 ± 5	~0.39	N.A.

* The uncertainty is obtained by taking the standard deviation of 18 independent measurements with one-minute averaging time. ** Dilution performed by injecting 20 mL of the calibrated source containing nine volatile species into a larger volume of 5.1 L N_2_ at atmospheric pressure.

## Supplementary Materials

The following are available online at https://www.mdpi.com/1424-8220/19/10/2334/s1, Figure S1: Experimentally measured (red curve) and simulated (blue curve) transmission spectra of methane. Figure S2: A structural overview of the gas handling system. Figure S3: An integrated gas handling system. Figure S4: A two-stage cooling system (water trap) including two cooling chambers. Figure S5: Temperature dependent vapor pressure of water and various other species. Figure S6: Absorbance spectrum of 5-ppmv ethanol and the obtained global fit representing a concentration of 4.87 ppmv. Figure S7: Summary of the concentration values obtained from a series of measurements involving four different pear-storage containers.
