# Supplementary material for: A Broadband Mid-Infrared Trace Gas Sensor Using Supercontinuum Light Source: Applications for Real-Time Quality Control for Fruit Storage
Source: Sensors (Basel). 2019 May 21;19(10):2334. doi: 10.3390/s19102334 (PMC6566869; doi:10.3390/s19102334)
Supplement: Supplementary file 1 [file sensors-19-02334-s001.pdf]

sensors

Article

A Broadband Mid-Infrared Trace Gas Sensor Using Supercontinuum Light Source: Applications for Real-Time Quality Control for Fruit Storage

Khalil Eslami Jahromi [1], Qing Pan [1,*], Amir Khodabakhsh [1], Cor Sikkens [1], Paul Assman [1], Simona M. Cristescu [1], Peter M. Moselund [2], Maxime Janssens [3], Bert E. Verlinden [3], and Frans J.M. Harren [1]

[1] Trace Gas Research Group, Institute of Molecules and Materials, Radboud University, 6525 AJ Nijmegen, The Netherlands; kh.eslami@science.ru.nl (K.E.J.); A.Khodabakhsh@science.ru.nl (A.K.); C.Sikkens@science.ru.nl (C.S.); p.assman@science.ru.nl (P.A.); s.cristescu@science.ru.nl (S.M.C.); F.Harren@science.ru.nl (F.J.M.H.)

[2] NKT Photonics A/S, Blokken 84, DK-3460 Birkeroed, Denmark; pmm@nktphotonics.com

[3] Flanders Center of Postharvest Technology, Willem de Croylaan 42, 3001 Heverlee, Belgium; maxime.janssens@vcbt.be (M.J.); bert.verlinden@vcbt.be (B.E.V.)

* Correspondence: qing.pan@science.ru.nl

Received: 24 April 2019; Accepted: 17 May 2019; Published: 21 May 2019

Supplementary Information

For completeness, this Supplementary Information includes additional information supporting the discussion of the main text.

Figure S1 shows the measured and simulated transmission spectra of methane, indicating a ~3 cm^{-1} spectral resolution of the grating-based spectrometer. Note that the simulation is inverted for clarity.

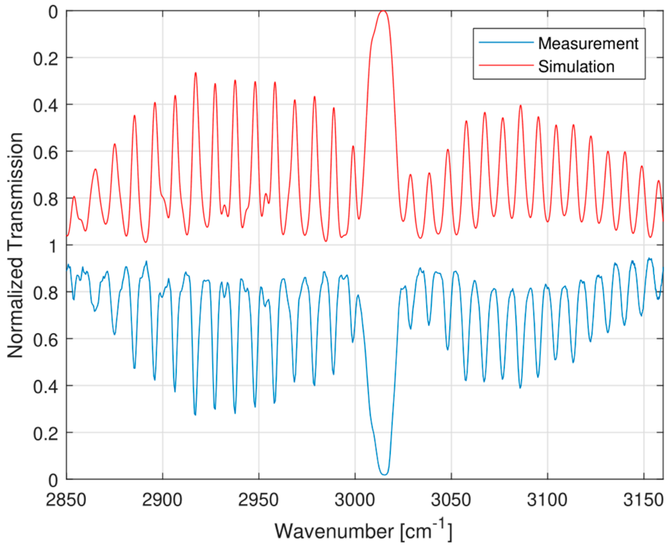

Figure S1. Experimentally measured (red curve) and simulated (blue curve) transmission spectra of methane.

Figure S2 shows the monitoring panel of the integrated gas handling system (HS: humidity sensor, MFC: mass-flow controller, PC: pressure controller). This system allows the detection of two samples (i.e. Sample #1 and Sample #2) in a closed loop configuration. An additional open loop is supported for Sample #3. The labels of A1, B1, etc., refer to two-way and three-way valves. The

catalyzer contains platinized Al_2O_3 pellets heated to 400 °C to convert nearly all the hydrocarbons to carbon dioxide and water. The associated hardware elements are shown in Figures S3 and S4.

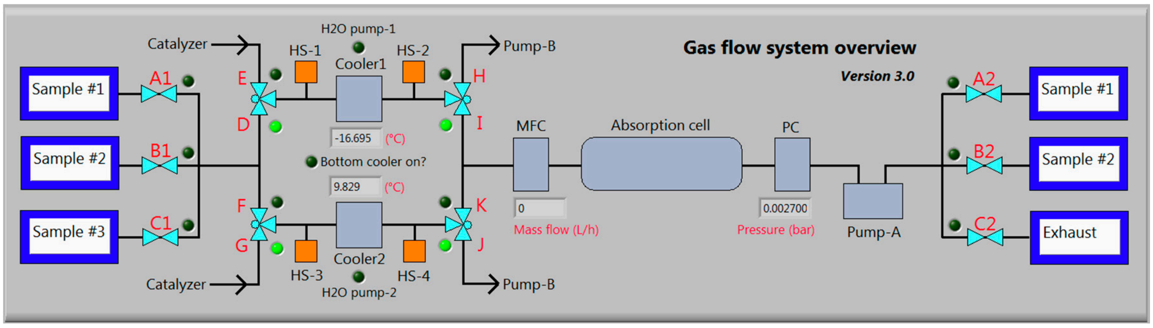

Figure S2. A structural overview of the gas handling system.

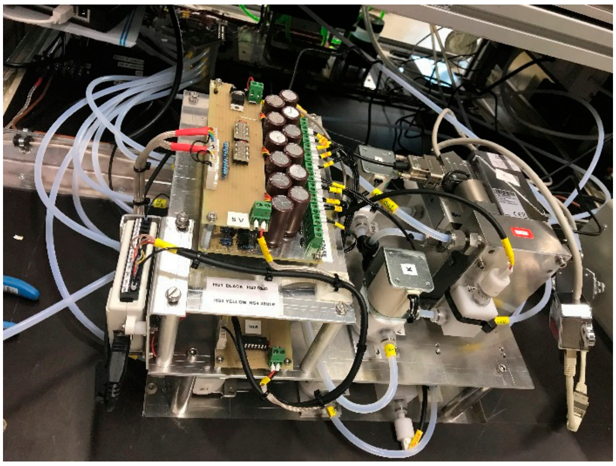

Figure S3. An integrated gas handling system.

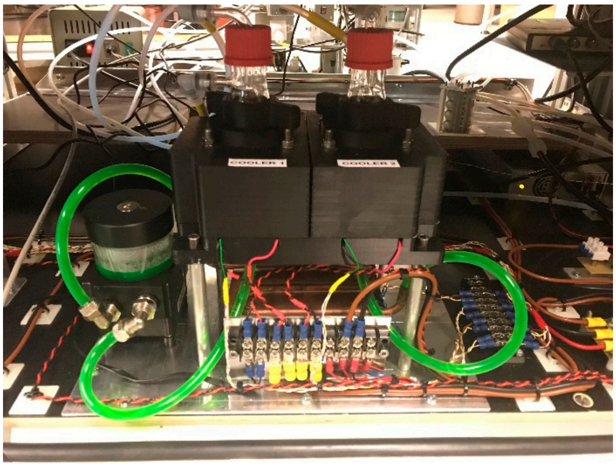

Figure S4. A two-stage cooling system (water trap) including two cooling chambers.

Figure S5 shows the calculated temperature dependent vapor pressure of various species. Note that water is considered as ice in contact with its own vapor. Since the vapor pressure of water is always lower than the vapor pressure of all the target species for the same temperature, cooling of the gas mixture is expected not to influence the phase of the target species significantly.

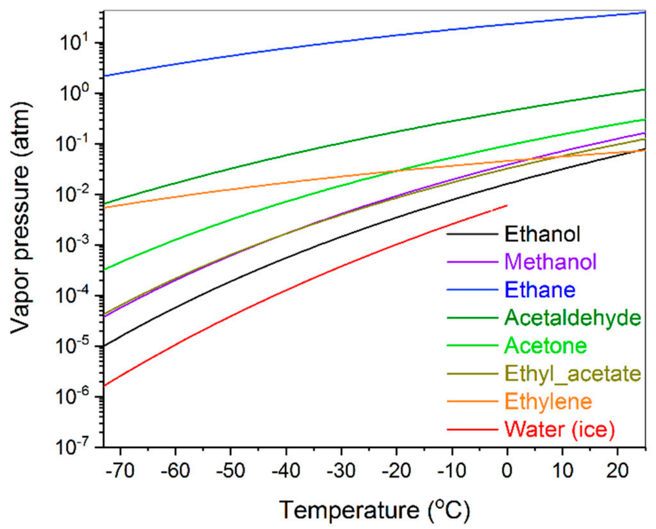

Figure S5. Temperature dependent vapor pressure of water and various other species.

Figure S6 shows a typical absorbance spectrum of 5 (±0.1) ppmv ethanol and the associated global fit representing 4.87 (±0.32) ppmv. The uncertainty is based on the standard deviation of 100 independent measurements. The peaks above 3000 cm^{-1} are related to water absorption. This result indicates that the adsorption effect due to the water trap (cooling chamber) is not significant.

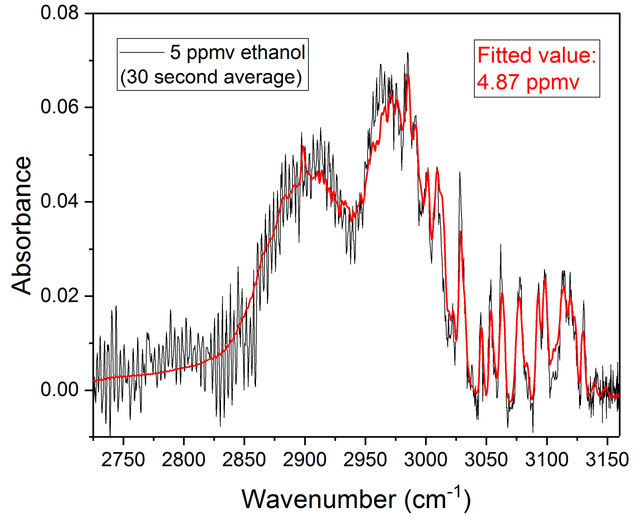

Figure S6. Absorbance spectrum of 5-ppmv ethanol and the obtained global fit representing a concentration of 4.87 ppmv.

Figure S7 summarizes a series of measurements performed on four different pear-storage containers. Each measurement contains 30–60 samples, each averaged for one minute. Each container has been measured twice in two consecutive weeks. The pears were harvested at the same time from the same orchard. The storage conditions are the following:

A: fixed O_2 of 2.5% and fixed CO_2 of 0.2%.

B: fixed O_2 of 2.5% and fixed CO_2 of 0.7%.

C: dynamically controlled atmosphere of 0.2% CO_2.

D: dynamically controller atmosphere of 0.7% CO_2.

The targeted gas species include the following: (a) ethanol, (b) methanol, (c) ethyl acetate, (d) acetone, and (e) acetaldehyde. Note that ethylene concentration is also monitored and presented. A reduced concentration contrast between ethylene and other volatiles tends to improve the accuracy of the measurements.

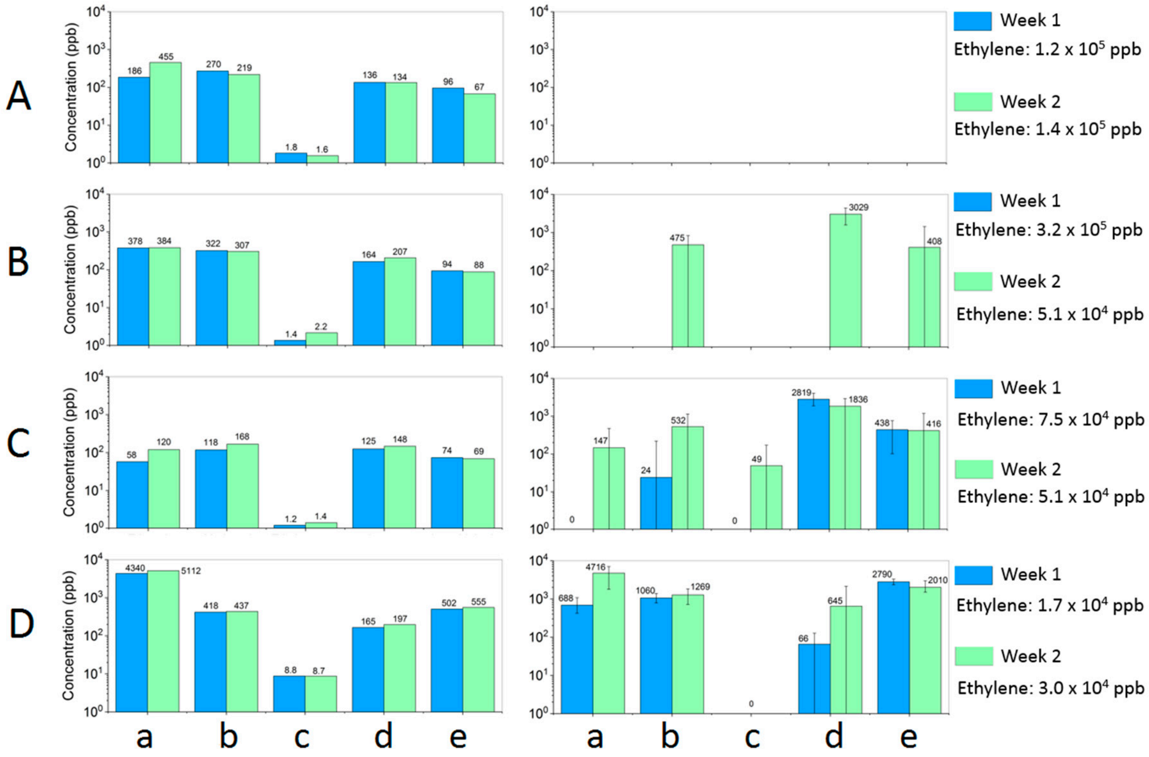

Figure S7. Summary of the concentration values obtained from a series of measurements involving four different pear-storage containers.
